# Urolithin A enhances muscle performance in elderly and positively impacts biomarkers linked to cellular health

**DOI:** 10.1093/geroni/igab046.2529

**Published:** 2021-12-17

**Authors:** Sophia Liu, David Marcinek

**Affiliations:** University of Washington, seattle, Washington, United States

## Abstract

Background: Aging is associated with decline in mitochondrial function and reduced exercise capacity. Urolithin A (UA) is a natural gut metabolite shown to stimulate mitophagy and improve muscle function in aged animals, and induce mitochondrial gene expression in elderly. Purpose: Investigate if oral administration of UA improved walking distance (6MWT), muscle fatigue resistance in hand (FDI) and leg (TA) muscles, and had an impact on plasma biomarkers. Method: We conducted a randomized, double-blind, placebo-controlled study (NCT03283462) in elderly subjects (65-90 yrs.) supplemented daily with 1000mg UA or placebo for 4 months. 128 subjects were screened and 66 randomized. 6MWT and ATPmax via MRS were assessed at baseline and at 4 months. Muscle fatigue tests and plasma analysis of biomarkers were assessed at baseline, 2 and 4 months. Results: UA significantly improved muscle endurance (i.e., change in number of muscle contractions from baseline) in two different muscles (hand: PL 11.6 ±147.5, UA 95.3 ± 115.5; and leg: PL 5.7± 127.1, UA 41.4 ±65.5) compared with placebo at 2-months. Plasma levels of several acylcarnitines, ceramides and C-reactive-protein were decreased by UA at the end-of study. 6MWT distance (PL 42.5 ± 73.3 m, UA 60.8± 67.2 m) and ATPmax increased in both groups from baseline (PL 13.7±31.4%, UA19.4± 56.8%) with UA supplemented group exhibiting greater improvements, although these were not statistically different between groups. Conclusion: UA supplementation improved muscle endurance, metabolic and inflammatory plasma biomarkers after 2-months, suggesting that UA can have a positive impact on muscle and cellular health in the elderly.

